# A direct sequencing assay for pharmacogenetic testing of thiopurine-intolerant *NUDT15* alleles in an Asian population

**DOI:** 10.1186/s13104-021-05821-3

**Published:** 2022-04-25

**Authors:** Kok-Siong Poon, Izz Irfan B. Imran, Silvester Kheng-Han Chew, Patrice Tan, Karen Mei-Ling Tan

**Affiliations:** 1 Department of Laboratory Medicine, National University Hospital, NUH Main Building, 5 Lower Kent Ridge Road, Singapore, 119074 Singapore; 2 School of Life Sciences and Chemical Technology, Ngee Ann Polytechnic, Singapore, Singapore

**Keywords:** *NUDT15*, Thiopurine, Pharmacogenetics, Direct sequencing

## Abstract

**Objective:**

The nucleoside diphosphate linked moiety X (Nudix)-Type motif 15 (NUDT15) enzyme is involved in thiopurine metabolism. Genetic variants in the *NUDT15* gene result in decreased NUDT15 activity, which in addition to decreased thiopurine S-methyltransferase (TPMT) activity, contributes to thiopurine toxicity. Current standard approaches of *NUDT15* genetic analysis have mainly been targeting several common variants. We aimed to develop a clinical-grade DNA-based assay for genetic analysis of the *NUDT15* gene using Sanger di-deoxy sequencing.

**Results:**

Sanger sequencing results were fully concordant with the expected *NUDT15* genotype in all 17 cell line samples with known *NUDT15* variants (accuracy = 100%; 95% CI 80.49 to 100.00%). Precision studies showed 100% intra-run repeatability and 100% inter-run reproducibility, respectively. Genetic analysis of the *NUDT15* gene was performed for 80 patients of Asian ethnicity with wildtype *TPMT*. 76% (N = 61) of the studied individuals had *NUDT15* *1/*1 diplotype. 25% (N = 14) of Chinese and 36% (N = 5) of Malays were found to carry at least 1 non-functional *NUDT15* allele. Our study confirmed a high frequency of *NUDT15* c.415C>T and c.55_56insGAGTCG variants in the Chinese and Malay ethnic groups in Singapore, highlighting the importance of determining *NUDT15* genotype prior to thiopurine dosing.

**Supplementary Information:**

The online version contains supplementary material available at 10.1186/s13104-021-05821-3.

## Introduction

Thiopurine drugs consist of azathioprine, 6-mercaptopurine, and thioguanine. These drugs are commonly prescribed to treat patients with acute lymphoblastic leukemias and autoimmune diseases such as inflammatory bowel disease (IBD) and rheumatoid arthritis. They are also used as adjunct immunosuppressive agents in solid organ transplantation. Thiopurines are prodrugs that are metabolized to the active 6-thioguanine nucleotide (6-TGN). The thiopurine S-methyltransferase (TPMT) enzyme metabolizes thiopurines to the inactive metabolite methylmercaptopurine, resulting in reduced active 6-TGN concentrations. The nucleoside diphosphate linked moiety X (Nudix)-Type motif 15 (NUDT15) enzyme hydrolyzes the active 6-TGN to the less active thioguanine monophosphate. Decreased TPMT and/or NUDT15 enzyme activities result in accumulation of active 6-TGN which causes myelotoxicity. Having a narrow therapeutic window, thiopurine drugs are known to be associated with a high frequency of adverse drug reactions (ADRs) [1, 2].

Dosing recommendations for thiopurine drugs based on *TPMT* genotype were published by the Clinical Pharmacogenetics Implementation Consortium (CPIC) a decade ago [3]. Subsequent landmark genome-wide association studies demonstrated strong association of the *NUDT15* c.415C>T variant with severe thiopurine-induced leukopenia and alopecia in Asians [4, 5]. Further studies in Asian children with leukemia [6, 7] and IBD patients [8, 9] demonstrated the utility of *NUDT15* c.415C>T variant analysis before administering thiopurines to avoid the adverse outcome of hematopoietic toxicity. These evidences led to the addition of *NUDT15* genotype to the latest CPIC guidelines for thiopurine dosing recommendations [10]. While TPMT activity can be tested using a biochemical assay or by genotyping, there is no available clinical biochemical assay for NUDT15 activity, indicating the importance of *NUDT15* genotype assays for the prescription of thiopurines.

**Fig. 1:**
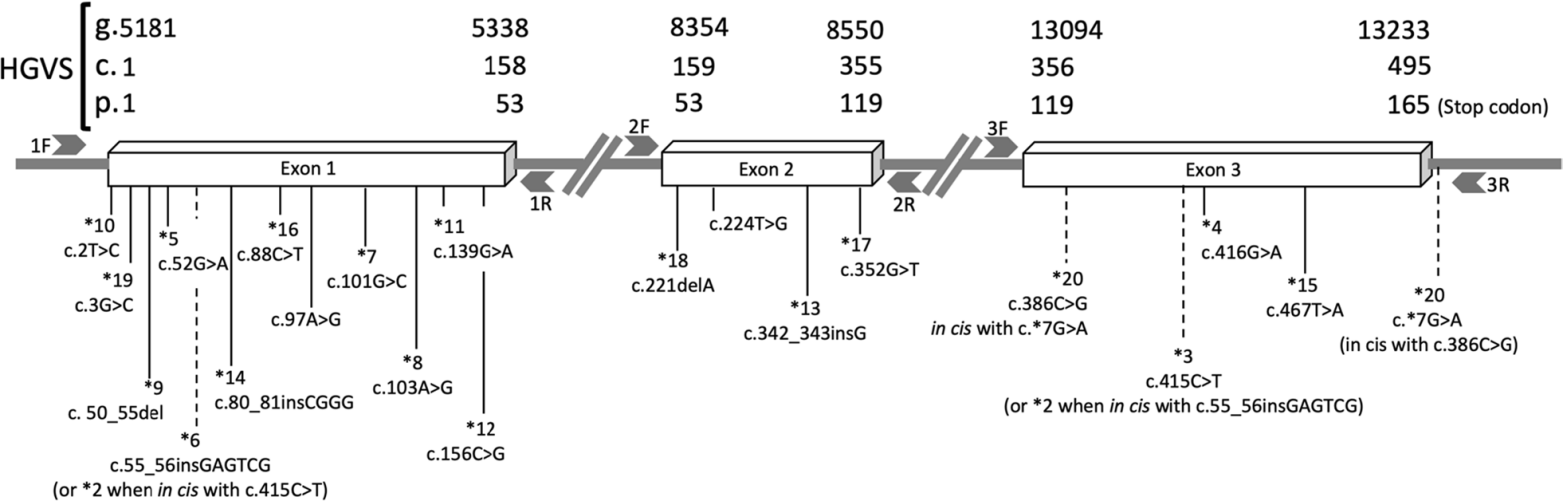
Schematic representation of the *NUDT15* gene with all known coding variants. The *2 to *20 alleles were reported in PharmVar. Arrows indicate PCR and sequencing primers (1F, 1R, 2F, 2R, 3F and 3R) flanking the three coding exons. Dotted vertical lines show alleles with two variants in cis. The *2 allele harbours two variants, namely c.55_56insGAGTCG (*6 in exon 1) and c.415C>T (*3 in exon 3). The *20 allele consists of c.386C>G in exon 3 and c.*7G>A in the 5′ unstranslated region. The c.97A>G and c.224T>G variants were reported in HGMD, but not in PharmVar [11]. HGVS, Human Genome Variation Society. Figure is not drawn to scale

Majority of *NUDT15* genotype assays target specific common variants, especially *3 (c.415C>T), *4 (c.416G>A), and *5 (c.52G>A) (https://www.mayocliniclabs.com/test-catalog/Clinical+and+Interpretive/65160, accessed on April 27, 2021; https://ltd.aruplab.com/Tests/Pub/3001535, accessed on April 27, 2021). Many *NUDT15* alleles (Fig. 1) have been described to date [10]. In this study, we developed a clinical-grade DNA-based assay for genetic analysis of the *NUDT15* gene using Sanger di-deoxy sequencing. We validated the assay using DNA from cell lines with known *NUDT15* variants. We further sequenced the *NUDT15* gene using this assay in a cohort of multi-ethnic Asian patients to determine the frequency of *NUDT15* variants.

## Main text

### Methods

#### Samples and DNA extraction

Genomic DNA extraction was performed on whole blood in EDTA-tubes using the LabTurbo Genomic DNA extraction kit (TAIGEN Bioscience Cooperation, Taipei, Taiwan) on the LabTurbo 48 Compact System (TAIGEN Bioscience Cooperation). Eighty patients with wildtype *TPMT* status by full gene sequencing were included in this study. Fifty-seven patients were Chinese, 14 were Malay and 9 were of other ethnicities. DNA samples from cell lines with known *NUDT15* variants used in this study (N = 17) were purchased from the Coriell Institute for Medical Research (New Jersey, United States).

#### Multiplex PCR and Sanger dideoxy sequencing assay

Three sets of primers (Additional file 1: Table S1) amplifying the partial 5′ untranslated region (UTR), three coding exons with immediate splice-sites and the partial 3′ UTR of the *NUDT15* gene (Fig. 1) were designed using the Primer3Plus online software (http://www.bioinformatics.nl/cgi-bin/primer3plus/primer3plus.cgi). Multiplex polymerase chain reaction (PCR) were performed in a 25 μL reaction with 1X HotStarTaq Master Mix (QIAGEN, Hilden, Germany), 0.4X Q solution (QIAGEN), 0.2 μM each of the forward and reverse primers (Integrated DNA Technologies, Singapore) and 100 ng of genomic DNA. Thermal cycling was performed on a SimpliAmp thermal cycler (Thermo Fisher Scientific, Waltham, USA) using the following conditions: initial denaturation at 95 °C for 5 min, followed by 35 cycles of 95 °C for 30 s, 62 °C for 30 s, and 72 °C for 45 s, followed by a final extension at 72 °C for 5 min. Three multiplex PCR products (593, 490 and 451 bp) were obtained. No non-specific bands were observed. The 3 PCR products were excised from the agarose gel and purified using the GeneAll Expin Kit (GeneAll Biotechnology, Seoul, Korea). The purified products were sequenced using the same forward and reverse PCR primers with the BigDye™ Terminator v3.1 Cycle Sequencing kit (Thermo Fisher Scientific). Sequencing products were purified using the DyeEx 2.0 Spin kit (QIAGEN) and capillary sequencing was performed on the Applied Biosystems 3500 Genetic Analyzer (Thermo Fisher Scientific).

#### Precision studies

DNA from the NA12878 cell line (wildtype *NUDT15*), and DNA from a whole blood sample harbouring heterozygous c.55_56insGAGTCG and heterozygous c.415C> T were sequenced in triplicates in the same sequencing run to assess intra-assay repeatability, and over three different sequencing runs on three different days to assess inter-assay reproducibility.

#### Variant analysis

Capillary electrophoresis data from the 3500 Genetic Analyzer was collected by the Data Collection software (Thermo Fisher Scientific) and analysed by the Sequencing Analysis software (Thermo Fisher Scientific). Nucleotide sequences were aligned against the genomic reference NG_047021.1 using ATF software (Conexio Genomics, Fremantle, Australia). Mutation Surveyor® (SoftGenetics, Pennsylvania, United States) was used to analyse and confirm the identified variants. Variant annotation was based on the mRNA reference NM_018283.3 and the protein reference NP_060753.1.

#### Statistical analysis

Statistical calculation of accuracy in this study was performed using an online statistical software, MEDCALC (https://www.medcalc.org/calc/diagnostic_test.php).

### Results

#### Analytical validity

Seventeen DNA samples from cell lines with known *NUDT15* variant status were sequenced in this study (Additional file 2: Table S2). Fifteen cell lines were previously characterised by 1000 Genomes phase 3. Of these 6 had heterozygous c.52G>A, 2 had homozygous c.415C>T, 4 had heterozygous c.415C>T and 3 had heterozygous c.416G>A. NA19240 and NA12878 cell lines were fully characterised and variant information are available from the Genetic Testing Reference Materials Coordination Program (GeT-RM) datasets. Sanger sequencing results matched 100% of the expected *NUDT15* genotype in all 17 cell lines (accuracy = 100%; 95% CI 80.49 to 100.00%). Precision studies performed on NA12878 with wildtype *NUDT*, and a DNA sample with c.55_56insGAGTCG and c.415C>T variants, showed 100% intra-run repeatability and 100% inter-run reproducibility.

#### Sanger sequencing results and *NUDT15* variant frequencies

**Fig. 2:**
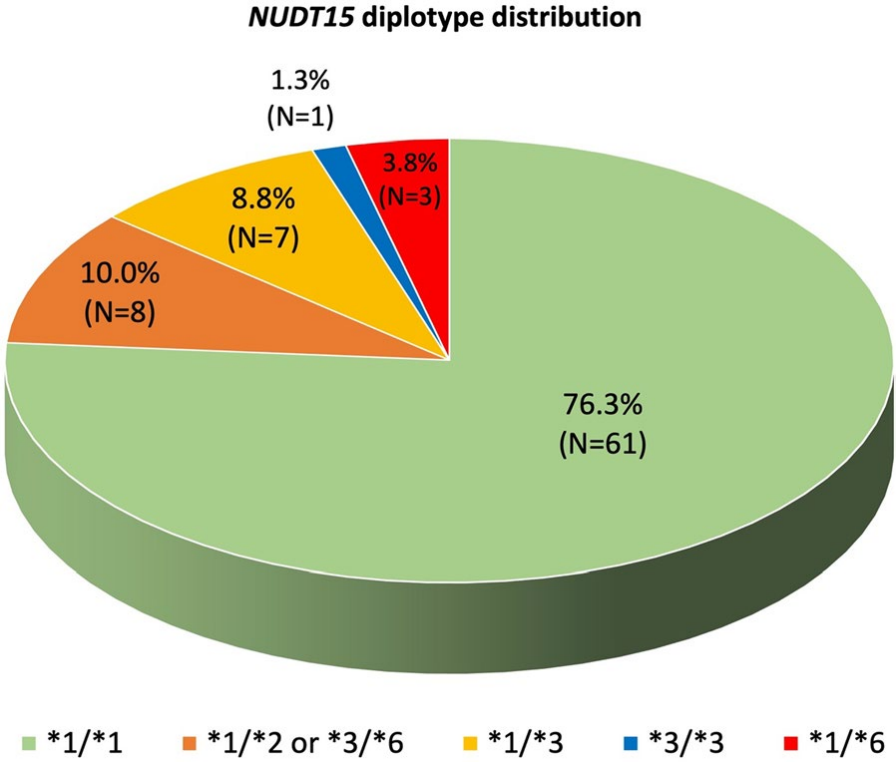
Pie chart shows the distribution of *NUDT15* diplotypes distributions in 80 individuals with *TPMT* *1/*1

In this study, *NUDT15* gene analysis was performed on DNA from 80 patients with wildtype *TPMT* status. Among all known *NUDT15* variants reported to date, only two were found in our studied samples, namely c.55_56insGAGTCG and c.415C>T. 76% (N = 61) of these studied individuals had *1/*1 diplotype while 24% (N = 19) had either *1/*2 (heterozygous c.415C>T and c.55_56insGAGTCG *in cis*), or *3/*6 (heterozygous c.415C>T and c.55_56insGAGTCG *in trans*), *1/*3 (heterozygous c.415C>T), *3/*3 (homozygous c.415C>T) or *1/*6 (heterozygous c.55_56insGAGTCG) diplotypes (Fig. 2). 25% (N = 14) of Chinese and 36% (N = 5) of Malays and were found to carry at least 1 thiopurine-intolerant *NUDT15* allele (Table 1). Out of 80 individuals sequenced, 61 (76%) were normal metabolizers, 18 (22.5%) intermediate metabolizers and 1 (1.25%) was a poor metabolizer. Among the Chinese, 42 (75%) were normal metabolizers, 13 (23%) were intermediate metabolizers and 1 (1.8%) was a poor metabolizer. Among the Malays, 9 (64%) were normal metabolizers and 5 (36%) were intermediate metabolizers.

**Table 1:** Distribution of *NUDT15* *1/*1 and non-*1/*1 diplotypes in individuals with TPMT *1/*1 in Singapore

Ethnicity	*NUDT15* diplotype	N =	Percentage (%)
Chinese	*1/*1	43	75.4
	Non-*1/*1	14	24.6
Sub-total		57	100
Malay (N = 14)	*1/*1	9	64.3
	Non-*1/*1	5	35.7
Sub-total		14	100
Others (N = 9)	*1/*1	9	100
	Non-*1/*1	0	0
Sub-total		9	100
All	*1/*1	61	76.2
	Non-*1/*1	19	23.8
Grand total		80	100

### Discussion

*NUDT15* genotyping is a clinical pharmacogenetic test which plays an important role in predicting thiopurine toxicity together with *TMPT* genotyping. *TMPT* genotyping has already been the standard test in this clinical realm since the recommendation made by CPIC in 2011 [3]. However, due to the relatively rare occurrence of *TPMT* risk alleles (*2, *3A, *3B and *3C) in Asian populations (1.4 to 2.5% from Moyer 2021) [12], the clinical utility of this test in patients of Asian descent is limited. A common *NUDT15* genetic variant, c.415C>T found in high frequency predominantly in East Asians [13] contributes to the genetic etiology of thiopurine-induced toxicity. Besides this common variant, other rare genetic variants in the *NUDT15* gene have also been reported [14, 15].

Since the discovery of *NUDT15* as a pharmacogenetic gene, multiple targeted approaches have been described including PCR-restriction fragment length polymorphism [16], high resolution melting [17, 18], pyrosequencing [19] and droplet digital PCR (ddPCR) [20]. Although these methods appear to be more economical and time-efficient, targeted assays may potentially miss rare variants that can only be detected by sequencing-based assays. Hence, a transition from approaches targeting specific *NUDT15* variants to sequencing the entire *NUDT15* gene for clinical testing was advocated in a recent review [12]. Sanger sequencing, the gold-standard method for DNA sequencing, requires on a deep understanding of the structure of gene of interest and the spectrum of genetic variants to design a good assay. However, it is not without its limitations. Sanger sequencing of PCR products is unable to differentiate two variants *in cis* on the same allele (e.g., *NUDT15* *1/*2) from compound heterozygosity of two variants *in trans* on two different alleles (e.g., *NUDT15* *3/*6). Tsujimoto et al. developed a method for differentiating the *NUDT15* *1/*2 and *NUDT15* *3/*6 diplotypes using ddPCR [20]. Recent advances in sequencing technologies using next-generation sequencing (NGS) approaches allow allelic phasing based on mapping of paired-end sequencing reads, enabling the differentiation between *NUDT15* *1/*2 and *NUDT15* *3/*6 diplotypes. Yu et al. developed a method using NGS to sequence messenger RNA (mRNA)-derived complementary DNA to determine *NUDT15* haplotypes [21], which was able to differentiate between *NUDT15* *1/*2 and *NUDT15* *3/*6 diplotypes. This method detects *NUDT15* variants on expressed transcripts, and serves as an “indirect” method for studying the genomic sequence of *NUDT15*. Using this approach, regulatory and intronic variants which potentially silence the normal *NUDT15* expression may be missed. Aberrantly spliced mRNA transcripts which can be difficult to be mapped on the reference mRNA transcript sequence, if encountered, still require confirmation of splice-site variants on genomic DNA. In addition to these caveats, mRNA transcripts with a truncating variant or premature stop codon are susceptible to nonsense-mediated decay and may probably be missed due to the limit of the assay’s sensitivity.

In this study, we developed a clinical direct sequencing assay for analyzing thiopurine-intolerant *NUDT15* alleles. We subsequently applied this method to study the *NUDT15* variant distribution in our local population. We found c.55_56insGAGTCG and c.415C>T, individually making up *3 and *6 alleles, or collectively named as *2 when appearing *in cis*, in a significant proportion of patients with wildtype *TPMT*. These two variants account for 100% of thiopurine-intolerant *NUDT15* alleles in our studied samples. The carriers of these variants are predicted to be at-risk of thiopurine-induced toxicity if a standard dose of thiopurine drugs was prescribed based on their *TPMT* status. According to gnomAD, the c.415C>T variant has a minor allele frequency (MAF) of 10.5% and 6.7% in East Asians and South Asians, respectively. The c.55_56insGAGTCG variant, on the other hand has 6.1% and 0.05% MAF in East Asians and South Asians, respectively. There is currently no MAF data for these two variants in the South East Asian populations.

In conclusion, we established a relatively simple and cost-effective clinical multiplex PCR with Sanger di-deoxy sequencing assay to detect *NUDT15* variants. Although this method cannot differentiate *NUDT15* *1/*2 and *NUDT15* *3/*6 diplotypes, it provides clinical information whether patients are wildtype or non-wildtype for *NUDT15* genotype, allowing clinicians to adjust the thiopurine dose for the treatment of leukemia and IBD to avoid adverse effects of myelotoxicity. Our study confirmed a high frequency of the c.55_56insGAGTCG and c.415C>T variants in Chinese and Malays in Singapore, and highlights the importance of determining *NUDT15* genotype status in addition to *TPMT* genotyping to predict thiopurine toxicity and aid in thiopurine dosing.

## Limitations

- The sample size in this study was small including 80 clinical samples and 17 cell line DNA.
- We limited our study cohort to only those individuals with wildtype *TPMT* genotype, as it is known that *TPMT* and *NUDT15* variants can co-exist. However, our study design serves to highlight the importance of *NUDT15* gene analysis in our population in addition to *TPMT* genotyping to predict thiopurine toxicity and aid in thiopurine drug dosing.
- The *NUDT15* *1/*2 and *NUDT15* *3/*6 diplotypes were not differentiated due to the technical limitation of Sanger sequencing in our study.

## Supplementary Information

**Additional file 1: Table S1.** List of primers for *NUDT15* PCR and sequencing analysis.

**Additional file 2: Table S2.** List of cell lines used to verify the Sanger sequencing assay. Data of expected *NUDT15* variants was retrieved from 1000 Genomes phase 3, except for NA19240 and NA12878, where the data were obtained from the Genetic Testing Reference Materials Coordination Program (GeT-RM) data set.

## Data Availability

All commercial cell line DNA are available at Coriell Institute for Medical Research (New Jersey, United States). All data generated during this study are included in this manuscript and in the supplementary tables.

## Abbreviations

ADRs: Adverse drug reactions
CPIC: Clinical Pharmacogenetics Implementation Consortium
MAF: Minor allele frequency
mRNA: Messenger RNA
NGS: Next-generation sequencing
*NUDT15*: Nucleoside diphosphate linked moiety X (Nudix)-Type motif 15
PCR: Polymerase chain reaction
*TPMT*: Thiopurine S-methyltransferase
UTR: Untranslated region
6-TGN: 6-Thioguanine nucleotides
