# Supplementary material for: A direct sequencing assay for pharmacogenetic testing of thiopurine-intolerant NUDT15 alleles in an Asian population
Source: BMC Res Notes. 2022 Apr 25;15:148. doi: 10.1186/s13104-021-05821-3 (PMC9036696; doi:10.1186/s13104-021-05821-3)
Supplement: Supplementary file 1 — Additional file 1: Table S1. List of primers for NUDT15 PCR and sequencing analysis. [file 13104_2021_5821_MOESM1_ESM.docx]

**Table S1** List of PCR and sequencing primers for *NUDT15* sequencing analysis.

| *NUDT15* gene region | Primers | Oligonucleotide sequences | PCR product size (base pairs) | Genomic coordinates (GRCh38.p12; Chr13) |
| --- | --- | --- | --- | --- |
| 5’UTR & exon 1 | 1F | CTGGGAGTGGATAGGCTGAC | 593 | 48037460 .. 48038052 |
|  | 1R | CAGAAGTCGAGGGGAGGAAC |  |  |
| Exon 2 | 2F | GCCCAGCTGATTTGTTGTTT | 490 | 48040719 .. 48041208 |
|  | 2R | GCATTCTCTTCATATGGCAACA |  |  |
| Exon 3 & 3’UTR | 3F | CAAGCAAATGCAAAGCATCA | 451 | 48045509 .. 48045959 |
|  | 3R | GGCTGAAAGAGTGGGGGATA |  |  |
