# Supplementary material for: A direct sequencing assay for pharmacogenetic testing of thiopurine-intolerant NUDT15 alleles in an Asian population
Source: BMC Res Notes. 2022 Apr 25;15:148. doi: 10.1186/s13104-021-05821-3 (PMC9036696; doi:10.1186/s13104-021-05821-3)
Supplement: Supplementary file 2 — Additional file 2: Table S2. List of cell lines used to verify the Sanger sequencing assay. Data of expected NUDT15 variants was retrieved from 1000 Genomes phase 3, except for NA19240 and NA12878, where the data were obtained from the Genetic Testing Reference Materials Coordination Program (GeT-RM) data set. [file 13104_2021_5821_MOESM2_ESM.docx]

**Table S2** List of cell line DNA used to verify the Sanger sequencing assay. Data of expected *NUDT15* variants was retrieved from 1000 Genomes phase 3, except for NA19240 and NA12878, of which the data was from Genetic Testing Reference Materials Coordination Program (GeT-RM) data set.

| No | ID | Gender | *NUDT15* variants identified by Sanger sequencing | | | Concordance |
| --- | --- | --- | --- | --- | --- | --- |
|  |  |  | c.52G>A | c.415C>T | c.416G>A |  |
| 1 | NA18535 | F | G/G | T/T | G/G | Yes |
| 2 | NA18599 | F | G/G | T/T | G/G | Yes |
| 3 | NA18619 | F | G/G | C/T | G/G | Yes |
| 4 | NA18620 | M | G/G | C/T | G/G | Yes |
| 5 | NA18621 | M | G/G | C/T | G/G | Yes |
| 6 | NA18622 | M | G/G | C/T | G/G | Yes |
| 7 | NA18566 | F | G/A | C/C | G/G | Yes |
| 8 | NA18567 | F | G/A | C/C | G/G | Yes |
| 9 | NA18615 | F | G/A | C/C | G/G | Yes |
| 10 | NA18997 | F | G/A | C/C | G/G | Yes |
| 11 | NA18997 | F | G/A | C/C | G/G | Yes |
| 12 | NA19079 | M | G/A | C/C | G/G | Yes |
| 13 | NA18940 | M | G/G | C/C | G/A | Yes |
| 14 | NA19740 | F | G/G | C/C | G/A | Yes |
| 15 | NA19746 | F | G/G | C/C | G/A | Yes |
| 16 | NA12878 | F | G/G | C/C | G/G | Yes |
| 17 | NA19240 | F | G/G | C/C | G/G | Yes |
